# Influence of Perineurial Cells and Toll-Like Receptors 2 and 9 on *Herpes simplex* Type 1 Entry to the Central Nervous System in Rat Encephalitis

**DOI:** 10.1371/journal.pone.0012350

**Published:** 2010-08-27

**Authors:** Biborka Bereczky-Veress, Nada Abdelmagid, Fredrik Piehl, Tomas Bergström, Tomas Olsson, Birgit Sköldenberg, Margarita Diez

**Affiliations:** 1 Department of Medicine Solna, Unit of Infectious Diseases, Karolinska Institutet, Karolinska University Hospital Solna, Stockholm, Sweden; 2 Department of Clinical Neuroscience, Neuroimmunology Unit, Karolinska Institutet, Karolinska University Hospital Solna, Stockholm, Sweden; 3 Department of Clinical Virology, University of Gothenburg, Göteborg, Sweden; Singapore Immunology Network, Singapore

## Abstract

*Herpes simplex* encephalitis (HSE) is a rare disease with high mortality and significant morbidity among survivors. We have previously shown that susceptibility to HSE was host-strain dependent, as severe, lethal HSE developed after injection of human *Herpes simplex* type 1 virus (HSV-1) into the whiskers area of DA rats, whereas PVG rats remained completely asymptomatic. In the present study we investigated the early immunokinetics in these strains to address the underlying molecular mechanisms for the observed difference. The virus distribution and the immunological responses were compared in the whiskers area, trigeminal ganglia and brain stem after 12 hours and the first four days following infection using immunohistochemistry and qRT-PCR. A conspicuous immunopathological finding was a strain-dependent difference in the spread of the HSV-1 virus to the trigeminal ganglia, only seen in DA rats already from 12 hpi. In the whiskers area infected perineurial cells were abundant in the susceptible DA strain after 2 dpi, whereas in the resistant PVG rats HSV-1 spread was confined only to the epineurium. In both strains activation of Iba1^+^/ED1^+^ phagocytic cells followed the distribution pattern of HSV-1 staining, which was visible already at 12 hours after infection. Notably, in PVG rats higher mRNA expression of Toll-like receptors (*Tlr*) *-2* and *-9*, together with increased staining for Iba1/ED1 was detected in the whiskers area. In contrast, all other *Tlr*-pathway markers were expressed at higher levels in the susceptible DA rats. Our data demonstrate the novel observation that genetically encoded properties of the host nerve and perineurial cells, recruitment of phagocyting cells together with the low expression of *Tlr2* and *-9* in the periphery define the susceptibility to HSV-1 entry into the nervous system.

## Introduction

*Herpes simplex* type-1 virus infects the majority of the population resulting in transient cold sores or non-symptomatic infection and then persists lifelong in the sensory ganglia of the infected individuals. Recurrent herpetic disease results from reactivation of HSV-1 from latency in sensory neurons and axonal transport to the periphery. Even though *Herpes simplex* virus (HSV) is a neurotropic virus, *Herpes simplex* encephalitis (HSE) occurs in only 2–3 previously healthy individuals/million/year in all age groups [1]. In more than ninety percent of the cases HSE is caused by HSV type1 and in the remaining by HSV type 2 [2]. HSE is characterized by acute onset of focal infection, inflammation and necrosis, mostly starting in the unilateral fronto-medio-basal temporal lobe. The disease has a tendency to relapse or to have a progressive course [3]. The mortality is high in the absence of treatment and there is significant morbidity in the survivors.

The ability of virus recognition by the innate mammalian immune system is critical in providing immediate antiviral effects mediated by type I interferons (α and β). These are produced within hours of infection and play a major role in the earliest host responses which inhibit virus replication in cells. The innate recognition of *Herpes* viruses involves several pathways. One is the detection of viral double stranded DNA by the Toll-like receptor (*Tlr*) *9* in the plasmacytoid dendritic cells (DC), which recognizes HSV-1 and -2. Dependent on the signaling cascade that is activated upon *Tlr9* binding, type I interferons or pro-inflammatory cytokines will be produced. Surface *Tlr2* on DCs and macrophages also bind virus glycoproteins and result in production of pro-inflammatory response [4]. This response can also be activated by the Interferon regulatory factor (*Irf*) *-7* transcription pathway.

The aim of the present study was to explore the early kinetics of the HSV-1 infection and the consecutive host-dependent innate immune responses after 12 hours and each of the first four days post infection (dpi) by utilizing a previously described DA rat model of lethal HSE [5]. This *in vivo* model largely resembles the human disease, starting from the whiskers area of the rats (the labio-facial area in humans). The virus penetrates the trigeminal nerve and subsequently the ipsilateral side of the brain stem, spreading there in contralateral and anterior direction. In our previous study, no traces of HSV-1 could be detected using qPCR, nor could live virus be retrieved using virus isolation in the trigeminal ganglia and the brain stem of the resistant PVG, while high titers were observed in the DA rats [5]. Our rat model is a model of acute encephalitis thus giving the possibility to investigate the early mechanisms underlying disease. By studying the development of the immediate immune responses and the molecular mechanisms occurring in the susceptible DA and the resistant PVG strains, important host regulated factors for the pathogenesis of HSV-1 may be revealed. Thus, in this study we have performed a detailed kinetic characterization of the virus infection in the DA and PVG strains which demonstrates distinct differences in the uptake of virus in the perineurium, recruitment of immune cells and expression of genes in the *Tlr*-pathway that underlie the observed difference in host strain susceptibility to HSE.

## Results

### Similar spread of HSV-1 in the whiskers area of DA and PVG at early time points, but further spread to the trigeminal ganglia detected only in DA rats

**Figure 1:**
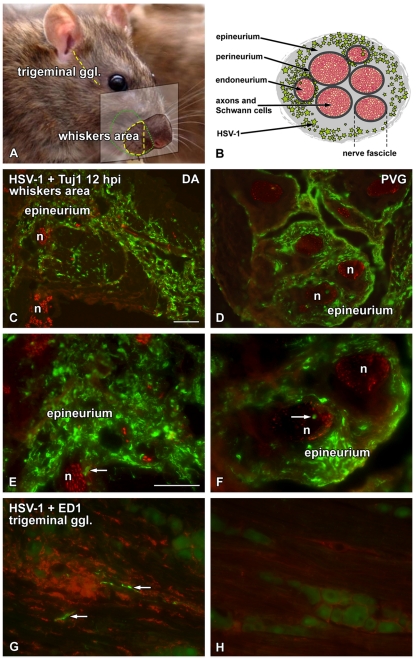
HSV-1 infected small nerve fascicles in both DA and PVG rats at 12 hpi. Schematic drawings and immunofluorescence micrographs illustrating the dissected site of infection and the viral spread in the whiskers area (A**–**F) and the trigeminal ganglia (A, G and H) at 12 hpi. The susceptible DA (C, E, G) and the resistant PVG (D, F, H) rat sections were stained with HSV-1 marker (green, C**–**H), neuronal marker Tuj1 (red, C**–**F) and phago-lysosome marker ED1 (red, G**–**H). Tissue from three animals at each time point were sectioned and processed for immunohistochemistry. (A) The panel presents the dissected part of the whiskers pad (green dotted line), the coronal/transversal section of the whiskers pad sliced onto the microscope glass (yellow dashed area) and the trigeminal ganglion, dissected from the base of the skull and sliced in sagittal/longitudinal direction (yellow dashed line). (B) Schematic picture of the cross-section of a peripheral nerve with HSV-1 propagation at 12 hpi in both DA and PVG rats. Virus could be observed in the vicinity of the smaller nerves with traces of HSV-1 positivity even in the endoneurium. (C - F) HSV-1 staining was observed in the immediate proximity of the nerve endings and the small nerve fascicles, even penetrating into occasional nerve fascicles in both DA and PVG rats. The epi- and perineurial cell layer of the larger nerve fascicles were uninfected in the whiskers area at this time point. (G and H) In the trigeminal ganglia of DA rats occasional HSV-1 staining was visible among the axons followed by infiltration of phagocyting ED1^+^ cells at 12 hpi, while in the trigeminal ganglia of the PVG rats no HSV-1 staining and sparse staining of ED1^+^ cells was detected. Scale bar: 50 µm.

HSV-1 was injected unilaterally into the whiskers area of two inbred rat strains, DA susceptible and PVG resistant to HSE. To investigate differences in viral spread, tissue response and immunological reaction, the whiskers area, the trigeminal ganglia (Figure 1A) and the brainstem at the level of trigeminal nerve entry were dissected and subsequently processed for staining using immunohistochemistry. Results are summarized in Table 1.

**Table 1:** Summary of inflammatory cell responses after HSV-1 infection in DA and PVG rats compared to controls.

		DA					PVG				
Area	Marker	12 hpi	1 dpi	2 dpi	3 dpi	4 dpi	12 hpi	1 dpi	2 dpi	3 dpi	4 dpi
**Whiskers area**	HSV-1	++	+	+++	+++	++++	++	+	+	+	+
	GFAP	-	-	+	+++	+++	-	-	-	-	-
	NKR	++	++	++	++++	++++	-	+	+	+	++
	Iba1	+	++	+++	+++	++++	+	++	++++	++++	++++
	ED1	+	++	++	+++	+++	++	+++	++++	++++	**+++**
	CD11b/c	-	+	+	++	++	-	+	+	++	++
	MHC I	+	++	+++	+++	+++	-	+	+	++	+++
	MHC II	-	-	+	+++	++++	-	-	+	++	+++
	CD8	-	+	+	++	+++	+	+	+	+	++
	HTX	+	++	+++	+++	++++	++	+++	++++	++++	+++
**Trigeminal ganglia**	HSV-1	+	+	+++	+++	++	-	-	-	-	-
	GFAP	-	-	-	+	++	-	-	-	-	-
	NKR	-/+	-/+	++	+++	++++	-	-	-	-	-
	Iba1	-	+	++	+++	+++	-	-	+	++	++
	ED1	++	++	++	++	+++	-	-/+	-/+	-/+	**-/+**
	CD11b/c	+	+	++	++	++	-	-	++	++	++
	MHC I	++	++	++	+++	+++	-	-	-	-	-
	MHC II	-	-	-	+	++	-	-	-	-	-
	CD8	-	-	++	++	++	-	-	-	-	-
	HTX	-	-	-	++	+++	-	-	-	-	-
**Brain stem**	HSV-1	-	-	-/+	+++	++++	-	-	-	-	-
	GFAP	-	-	-	++	+++	-	-	-	-	-
	NKR	-	-	-	+	+++	-	-	-	-	-
	Iba1	-	-	-	++	+++	-	-	-	-	-
	ED1	-/+	-/+	+	++	++++	-	-	-	-	**-**
	CD11b/c	-	-	+	++	+++	-	-	-	-	-
	MHC I	-	-	+	++	+++	-	-	-	-	-
	MHC II	-	-	-	+	++	-	-	-	-	-
	CD8	-	-	-	+	++	-	-	-	-	-
	HTX	-	-	-	+	++	-	-	-	-	-

(-) no difference, (-/+) traces of up-regulation, (+) small increase, (++) moderate increase, (+++) strong increase, (++++) wide-spread, very intense increase.

At 12 hours post-infection (hpi) the HSV-1 spread was similar in the whiskers area of DA and PVG rats. The virus was spread in the epineurium, *i.e.* the outermost layer of connective tissue surrounding several nerve fascicules/bundles of peripheral nerves and in the immediate vicinity of individual nerve fibers (Figure 1B) both in DA (Figure 1C and 1E) and PVG (Figure 1D and 1F) rats, as well as in cells within the perineurium, *i.e.* the layer of connective tissue surrounding the nerve fascicles (Figure 1B). In some nerve fascicles of both DA and PVG rats, virus staining was also found inside the nerve, either as clear staining or as traces of staining (Figure 1E–1F). However, in the resistant PVG strain, virus detection in the nerve was more occasional than in the DA. At 12 hpi virus was detected in the trigeminal ganglia of some individuals of the susceptible DA (Figure 1G), but none of the resistant PVG strain (Figure 1H). At 1 dpi HSV-1 staining at the infection site was interestingly decreased in both strains as compared to 12 hpi.

Macrophages and microglia specifically express Iba1, a calcium-binding protein, used as a marker of tissue inflammation and their phagocytic activity can be detected by staining for ED1 antibody, which recognizes a glycoprotein on the membrane of phago-lysosomes. The activation and recruitment of Iba1^+^/ED1^+^ cells to the site of infection was similar between DA and PVG (Table1), but most pronounced at 12 hpi in the outer parts of the epineurium and in the tissue surrounding the epineurium (Figure 1B). However, scattered positive cells were also found in the inner part of epineurium and in the perineurium. More recruitment of ED1^+^ cells to the outer part of epineurium was seen in PVG compared to DA rats (Table 1). Iba1^+^/ED1^+^ cells were also seen inside some nerve fascicles of both strains, mostly in large nerves of DA rats. At 1 dpi more immune cells were recruited to the inner part of the epineurium in both strains, even though many cells were still in the outer part of the epineurium.

The staining for major histocompatibility complex class I (MHC I) was higher around small nerve fascicles and in the perineurium of DA rats compared to PVG. Staining was also observed in the endoneurium, *i.e.* the layer of connective tissue surrounding each nerve fiber inside the nerve fascicle (Figure 1B) of both strains, but to a higher extent in DA rats (Table 1).

### HSV-1 infected perineurial cells in the whiskers area of DA, but not PVG rats

**Figure 2:**
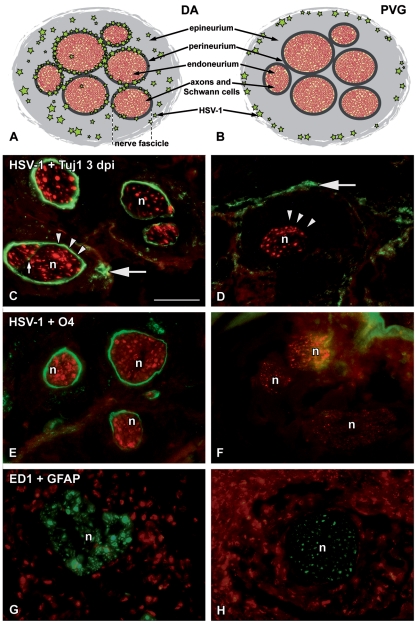
HSV-1 infected perineurium and changed GFAP expression in the whiskers area of susceptible DA rats. Schematic drawings of the peripheral nerve structure in the whiskers area and the localization of the HSV-1 staining (A and B) and immunofluorescence micrographs showing sections of the whiskers area at 3 dpi (C**–**H). The DA (A, C, E, G) and the PVG (B, D, F, H) sections were stained with HSV-1 marker (green, C**–**F), neuronal marker Tuj1 (red, C and D), oligodendroglia marker O4 (red, E and F), phago-lysosome marker ED1 (red, G and H) and Schwann cell marker GFAP (green, G and H). (A, C and E) In DA rats increased HSV-1 staining (green) was observed in the perineurial cell layer (arrowheads in C) surrounding nerve fascicles stained with the neuronal marker Tuj1 (red) and oligodendrocyte marker O4 (red) at 3 dpi. HSV-1 staining was also found in the epineurium (large arrow in C) and at increased level in the endoneurium (small arrow in C). (B, D and F) In PVG rats no HSV-1 staining was visualized in the perineurial cell layer at 3 dpi (arrowheads in D), instead HSV-1 was strictly confined to the outer part of the epineurium (large arrow in D). (G and H) Schwann cells stained with GFAP (green) displayed an altered morphology in the nerve fascicles of the DA rats at 3 dpi, a finding not present in PVG rats. Green stars represent HSV-1; arrowheads indicate the perineurial cell layer; large arrows indicate HSV-1 staining in the epineurium; small arrow indicates HSV-1 staining in the endoneurium; n, nerve fascicle. Scale bar: 50 µm.

**Figure 3:**
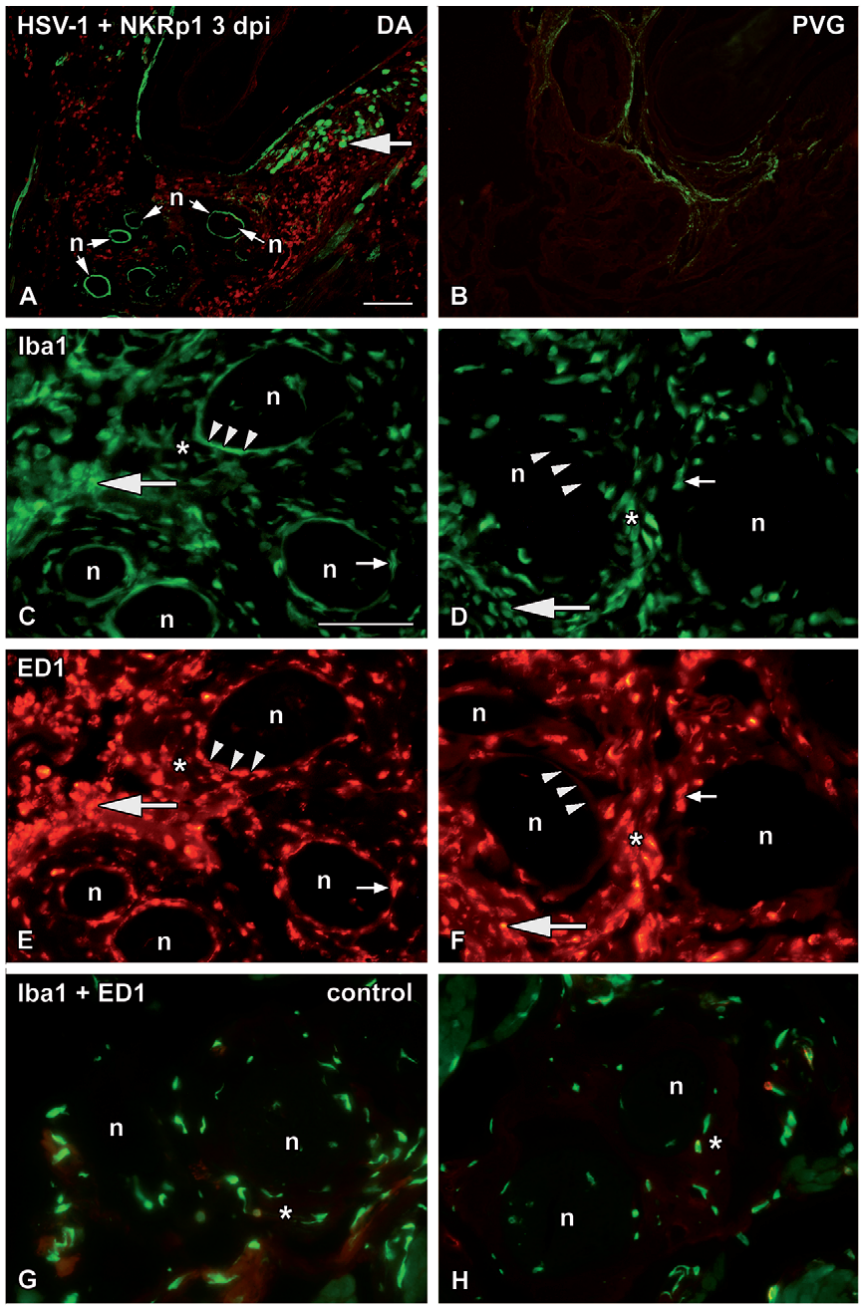
Differences in the recruitment of NK and phagocytic cells to the epi- and perineurium. Immunofluorescence micrographs illustrating sections of the whiskers area at 3 dpi (A**–**F) and controls (G**–**H). The DA (A, C, E, G) and PVG (B, D, F, H) sections were stained with HSV-1 marker (green, A and B), NK-cell marker (red, A and B), macrophage marker Iba1 (green, C, D, G and H) and phago-lysosome marker ED1 (red, E, F, G and H). (A and B) Massive infiltration of NK cells (red) and HSV-1 staining surrounding the nerve fascicles was seen in the susceptible DA rats, whereas in the resistant PVG only sparse NK cells and no perineurial HSV-1 staining was seen at the site of the infection. In DA rats HSV-1 also infected muscle fibers (large arrow in A), but not in PVG. (C and E) In DA rats, Iba1^+^ and ED1^+^ cells were detected in the perineurial cell layer (arrowheads) in the same pattern as HSV-1 staining. (D and F) In PVG rats, only occasional Iba1^+^ and ED1^+^ cells (small arrows) were found in the perineurial cell layer (arrowheads) of the nerves. (C–F) Extensive infiltration of Iba1^+^/ED1^+^ cells was seen in the epineurium of both DA and PVG rats (large arrows), although slightly less in DA (indicated by asterisk) when compared to PVG rats. (G and H) Scattered Iba1^+^ and ED1^+^cells were seen in DA and PVG control rats in similar distribution. Asterisks indicate the inner part of the epineurium; large arrows indicate the outer part of the epineurium; small arrows point to Iba1^+^/ED1^+^ co-localization; arrowheads point to staining in the perineurium; n, nerve fascicle. Scale bar: 50 µm.

After 1 dpi, at all time points HSV-1 was more spread in the susceptible DA, compared to the resistant PVG rats (Figure 2A–2F). The most striking finding was the intense, ring-formed staining of HSV-1 in the perineurium, observed only in DA rats (Figure 2A, 2C, 2E and 3A) from 2 dpi, increasing at later time points. From 3 dpi the staining was widespread in the epi- and perineurium of the DA rats, while in the endoneurium only traces of staining were observed (Figure 2C, small arrow).

In the whiskers area of PVG rats no HSV-1 staining was detected in the peri- and endoneurium from 2 dpi, but virus remained at all time points in the outer part of the epineurium (Figures 2B and 2D).

In the trigeminal ganglia of DA rats staining for HSV-1 increased from 2 dpi, while in the brain stem it was first detected at this time point. In PVG rats no virus was detected at any time point in these two compartments (Table 1).

### Changed morphology of Schwann cells in DA rats

To investigate the effect of HSV-1 infection on Schwann cells in peripheral nerves and on astrocytes in the CNS we used antibodies against oligodendroglia marker (O4) and Glial fibrillary acidic protein (GFAP). In the whiskers area O4 staining (Figure 2E and 2F) was seen in the same compartment as where the GFAP staining was seen (Figure 2G and 2H). GFAP staining inside the nerves was modified from 2 dpi in DA rats (Figure 2G), indicating changed morphology of the Schwann cells due to the virus infection, which was not observed in PVG rats (Figure 2H). In the trigeminal ganglia and the brain stem up-regulation of GFAP positive staining was visible from 3 dpi. No up-regulation of GFAP was detected in PVG rats in any compartment (Table 1).

### Early recruitment of NK and CD8^+^ cells in DA rats

More NK cells were visible in the whiskers area of the DA compared to PVG rats from 12 hpi, increasing after 2 dpi in DA rats (Figure 3A and 3B and Table 1). In the DA rats infiltrating NK cells outnumbered the CD8^+^ T-cells from 12 hpi and onwards.

In the trigeminal ganglia and the brain stem of DA rats, NK cells and CD8^+^ cells were seen from 2 and 3 dpi, respectively. In the PVG rats no NK or CD8^+^ cells were seen in the trigeminal ganglia or the brain stem at any time point (Table1).

### Activation of macrophages and dendritic cells after HSV-1 infection

Infiltration and activation of macrophages (Iba1 and complement receptor 3 CD11b/c markers) with phagocytic activity (ED1 marker) in the whiskers area was further increased at later time points, *i.e.* after 1 dpi (Table 1).

After HSV-1 infection, more Iba1^+^ cells than CD11b/c^+^ cells were observed in the whiskers area in both strains. In spite of the increased immune cell infiltration in both DA and PVG rats, the distribution differed in the epi- and perineurium (Figure 3C, 3D and Table 1). In DA rats Iba1^+^ and ED1^+^ cells delineated the perineurium (arrowheads in Figure 3C and 3E), a finding which was not seen in PVG rats (arrowheads in Figure 3D and 3F). In both DA and PVG rats most Iba1^+^ cells were also staining for ED1 (small arrows in Figure 3C–3F), indicating phagocytic activity. In the whiskers area of both DA and PVG controls, Iba1^+^ cells were also detected to a much lower extent (Figure 3G and 3H).

**Figure 4:**
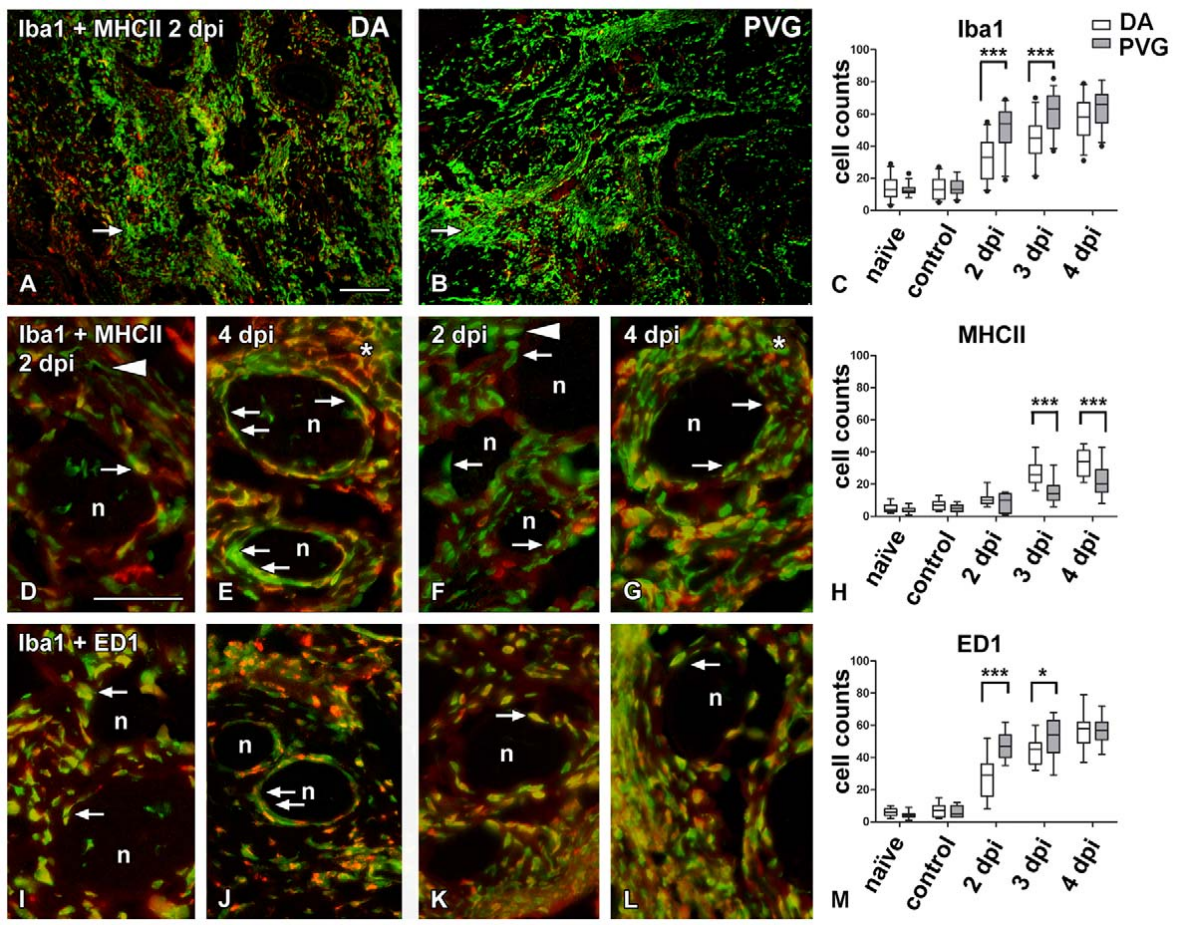
Delayed recruitment of phagocytic cells and increased MHC II activation in the epineurium of DA rats. Immunofluorescence micrographs illustrating sections of the whiskers area at 2 dpi (A, B, D, F, I and K) and 4 dpi (E, G, J and L). The DA (A, D, E, I and J) and the PVG (B, F, G, K and L) sections were stained with macrophage marker Iba1 (green, A, B, D**–**G and I**–**L), MHC II marker (red, A, B and D**–**G) to show the antigen presenting properties and phago-lysosome marker ED1 (red, I**–**L). Cellular quantification is represented in C, H and M panels. (A**–**C) At 2 dpi massive recruitment of Iba1^+^ cells (green) was present in the whiskers area of both DA (A) and PVG (B) rats at the site of the infection. However, the quantification of infiltrating Iba1^+^ cells to the epineurium (C) showed that the recruitment of macrophages was delayed and reduced in the epineurium of DA (arrow in A) compared to PVG rats (arrow in B). (D**–**H) At 2 dpi (D, F, H) the activation of antigen presenting cells detected by MHC II (red) was similar in both strains, but at 3 dpi (H) and 4 dpi (E and H) MHC II increased in DA compared to PVG rats (G and H). At 4 dpi in the whiskers area of DA rats (E) Iba1^+^/MHC II^+^ cells (arrows in E) were seen in the perineurium, in a pattern similar to the HSV-1 staining seen in Figure 1. In PVG rats (G), double and single stained Iba1^+^ and MHC II^+^ cells were more scattered in the vicinity of nerve fascicles (arrows in G), not delineating the perineurium in the same way as in DA. (I**–**M) Most Iba1^+^ cells were also ED1^+^ in both DA (I and J) and in PVG rats (K and L) indicating their phagocytic activity (arrows). However, there were more ED1^+^ cells in PVG at 2 dpi and 3 dpi compared to DA (M). At 4 dpi (J and L), Iba1^+^/ED1^+^ cells clearly delineated the perineurial cell layer in DA (arrows in J), but not in PVG (arrow in L), where these cells remained in the epineurium. Asterisks (E and G) point to the cellular infiltration into the epineurium; small arrows indicate cells in the perineurium; arrowheads indicate differences in Iba1^+^ morphology in DA compared to PVG rats; n, nerve fascicles; dots in panel C represent outliers. Scale bar: 50 µm.

In the whiskers area of DA rats from 2 dpi there was lower and delayed activation of Iba1^+^ (Figure 4C) and ED1^+^ (Figure 4M) (asterisk in Figure 3C, 3E, 4E), as well as of CD11b/c^+^ cells (Table1) compared to the PVG rats (asterisk in Figure 3D and 3F), despite the fact that more HSV-1 staining was visible in this area in DA rats. The differences were seen both visually and by counting infiltrating immunopositive cells in the inner part of the epineurium (Table 1, Figure 4C and 4M).

In the trigeminal ganglia of the DA rats, ED1^+^ and CD11b/c^+^ staining was up-regulated from 12 hpi, with further increase at 2 dpi (Figure 1G and Table 1). Interestingly, in PVG at 2 dpi Iba1^+^, ED1^+^ and CD11b/c^+^ staining was seen in the absence of HSV-1, possibly indicating a retrograde reaction and/or systemic involvement.

In the brain stem of DA rats, tissue activation and immune cell infiltration were seen from 2 dpi correlating with the HSV-1 staining, which was not detected in the resistant PVG rats (Table 1).

### Major histocompatibility complex (MHC) class I and II expression followed the immune cell distribution

To further explore the cellular activation caused by HSV-1 infection and its influence on antigen presentation in DA and PVG rats, we stained for MHC I and MHC II.

In the whiskers area MHC I up-regulation started in DA rats from 12 hpi, whereas in the PVG it was increased from 1 dpi reaching the same level as in DA rats at 4 dpi (Table 1).

The activation of MHC II was delayed compared to MHC I, starting from 2 dpi. Most MHC II^+^ cells in the whiskers area were also Iba1^+^ cells (Figure 4D–4G) in both strains. Even though there was lower infiltration of Iba1^+^ cells in DA, these cells express MHC II at a higher degree compared to PVG (Figure 4C and 4H, Table 1). Iba1^+^/MHC II^+^ cells were concentrated both in the epi- and perineurium in DA (Figure 4D and 4E), but in PVG rats only in the epineurium (Figure 4F and 4G). Notably, in PVG rats the number of Iba1^+^/ED1^+^ cells was higher in the whiskers area (Figure 4C, 4I–4M), however the MHC II expression was lower compared to the susceptible DA strain (Figure 4H), suggesting increased phagocytic activity in the resistant PVG compared to the susceptible DA rat and possibly decreased antigen presentation capacity (Figure 4I–4L).

In the trigeminal ganglia of DA rats up-regulation of MHC I was seen from 12 hpi, and MHC II from 3 dpi, whereas in the brain stem level of MHC I expression was increased from 2 dpi and level of MHC II expression from 3 dpi. No up-regulation of MHC I or II was visible in the trigeminal ganglia or the brain stem of the PVG rats (Table 1).

### Hematoxylin-eosin staining revealed early recruitment of immune cells in PVG rats

In both strains cellular infiltration was mainly seen in the whiskers area, although cell numbers were higher in PVG rats. In contrast to DA, no cellular infiltration was seen in the trigeminal ganglia or in the brain stem of PVG rats (Table 1).

### mRNA expression of key molecules in the Toll-like receptors (*Tlr*) pathway

**Figure 5:**
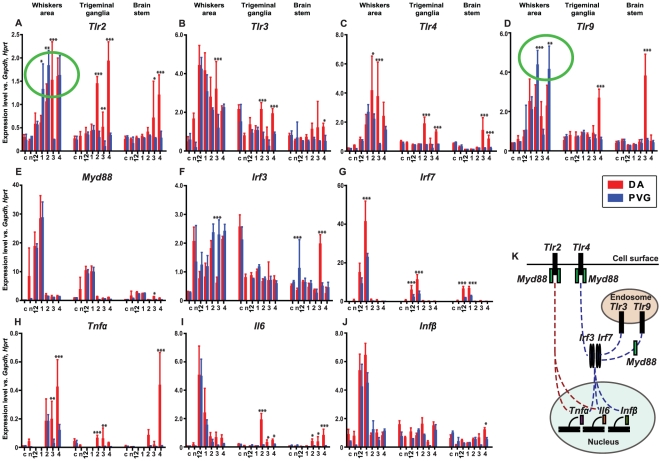
Host-dependent regulation of molecules in the *Tlr* signaling pathway after HSV-1 infection. Quantitative evaluation of mRNA expression levels after HSV-1 infection in the whiskers area, the trigeminal ganglia and the brain stem of infected susceptible DA, resistant PVG and in control rats and at different time points after infection. Measurements were made in 5 individuals at each time point studied and all measurements were done in triplicates. Values were compared to the expression levels of the housekeeping genes: *Gapdh* and *Hprt*. (A) The expression level of *Tlr2* gene after HSV-1 infection was significantly lower in the susceptible DA rats in the whiskers area at 1 and 2 dpi. In the trigeminal ganglia induction was seen in DA rats from 2 dpi and in the brain stem from 3 dpi. (B) *Tlr3* expression level was significantly higher in DA rats at 3 dpi in the whiskers area, at 2 and 4 dpi in the trigeminal ganglia and in the brain stem at 4 dpi compared to PVG rats. (C) Significantly increased expression level of *Tlr4* was seen in DA rats at 2 and 3 dpi in the whiskers area, at 2 and 4 dpi in the trigeminal ganglia and at 3 and 4 dpi in the brain stem as compared to the PVG rats. (D) The *Tlr9* expression level was significantly lower in the whiskers area of the DA rats at 2 and 4 dpi. In contrast, expression level was higher in DA rats in the trigeminal ganglia at 4 dpi and in the brain stem at 3 dpi. (E) No difference was seen in the expression level of the adaptor molecule *Myd88* between DA and PVG rats, except for an induction in the brain stem of DA rats at 3 dpi. (F) In the resistant PVG strain, the transcription factor *Irf3* was induced in the whiskers area at 3 dpi and interestingly in the brain stem of naïve rats compared to DA. In the DA rats expression was induced in the brain stem at 3 dpi. (G) The transcription factor *Irf7* was significantly induced in DA rats compared to the PVG rats at early time points; in the whiskers area at 1 dpi, in the trigeminal ganglia and the brain stem at 12 hpi and 1 dpi. (H) The pro-inflammatory cytokine *Tnfα* expression was induced in the DA rats compared to the PVG rats in the whiskers area at 3 and 4 dpi, the trigeminal ganglia at 2 and 3 dpi and the brain stem at 4 dpi. (I) The *Il6* pro-inflammatory cytokine response was similar in the whiskers area of both strains, but in the DA rats the expression was induced in the trigeminal ganglia and the brain stem from 2 dpi. (J) No difference was seen in the *Ifnβ* response between DA and PVG rats, except for higher expression in DA rats in the brain stem at 4 dpi. (K) Simplified illustration of the Toll-like receptor signaling cascade including the molecules measured in this study. Abbreviations: c - controls, n - naïve, 12 - 12 hpi, 1 - 1 dpi, 2 - 2 dpi, 3 - 3 dpi and 4 - 4 dpi. The green circles (A and D) highlight the significant differences between DA and PVG rats in *Tlr2* and -*9* expression in the whiskers area. Error bars represent the standard deviation for the five individuals included at each time point. P value: *** ≤0.001, ** ≤0.01, * ≤0.05.

To investigate the contribution of *Tlr*-pathway to the activation of innate immune cells in HSE, we assessed the change in mRNA expression of key molecules within this pathway (Figure 5K) after infection using qRT-PCR in the same compartments studied as with immunohistochemistry, *i.e*. the whiskers area, the trigeminal ganglia and the brain stem of susceptible DA and resistant PVG rats (Figure 1A).

Toll-like receptors are conserved innate receptors expressed in immune and non-immune cells. *Tlr2* and -*9* are the most important in recognizing HSV-1 glycoproteins and double-stranded DNA, respectively [4]. All *Tlrs* signal through the adaptor protein Myeloid differentiation primary response factor (*Myd88*), except for *Tlr3*; then the transcription factors, Nuclear factor kappa B (NFκB), Interferon regulatory factor (*Irf*) 3 and 7 leading to the induction of effector pro-inflammatory cytokines *Tnfα* and *Il6* as well as type I inteferons *Ifnβ* (Figure 5K).

In both strains, the highest change in expression levels of early inflammatory markers was in the whiskers area, compared to the trigeminal ganglia and the brain stem (Figure 5). These findings point towards the importance of the innate immune responses in the periphery for HSE development in our model.

There were two patterns of expression of the innate molecules: one with continuously increasing level from 12 hpi, *i.e. Tlr2 and -9*, *Tnfα*; and the other with the highest expression level between 12 hpi and 1 dpi followed by gradual decrease, *i.e. Tlr3*, *Myd88*, *Irf7*, *Ifnβ* and *Il6*.

### Low *Tlr2* and *Tlr9* mRNA expression in the susceptible DA rats

*Tlr2*, *Tlr4*, *Tlr9*, *Irf7* and *Tnfα* were the molecules which differed most between the susceptible and the resistant strains (Figure 5A, 5C, 5D, 5G and 5H). *Tlr4*, *Irf7* and *Tnfα* were increased in the DA rats (Figure 5C, 5G and 5H), possibly reflecting the spread of HSV-1 and the infiltration of the NK cells in this strain visualized by immunohistochemistry (Figure 3A and 3B). Notably, the levels of *Tlr2* and *-9* were higher in the resistant PVG rats (Figure 5A and 5D). This might relate to the earlier recruitment of phagocyting cells (Figure 4C and 4M). Interestingly, DA rats presented lower expression of *Tlr2* in the whiskers area at 1–2 dpi (Figure 5A) and *Tlr9* from 2 dpi (Figure 5D), correlating with a lower recruitment of phagocyting cells in this compartment (Figure 4C and 4M).

In the whiskers area of both strains the highest levels of expression were detected for *Irf7* at 1 dpi. In DA rats the expression was induced to twice the level of PVG and a hundred-fold compared to the naïve (Figure 5G). *Irf7* was also increased in the trigeminal ganglia and the brain stem at 12 hpi and 1 dpi in both strains, suggesting a systemic response to the viral infection, as the virus had not yet reached these compartments (Figure 5G).

In the trigeminal ganglia and the brain stem most targets showed higher expression levels in the DA compared to the PVG rats, which displayed no or only modestly elevated levels of the studied markers (Figure 5A–5J).

### Kinetics of expression of inflammatory mediators

The levels of significance for change in mRNA expression of key molecules in the *Tlr*-pathway over time are presented in Table 2.

**Table 2:** mRNA expression differences of innate immune markers after HSV-1 infection compared to naïve tissue.

		DA					PVG				
Area	Target	12 hpi	1 dpi	2 dpi	3 dpi	4 dpi	12 hpi	1 dpi	2 dpi	3 dpi	4 dpi
**Whiskers area**	*Tlr2*	*ns*	*ns*	<0.05	<0.01	<0.001	*ns*	<0.001	<0.001	*ns*	<0.001
	*Tlr3*	<0.001	<0.01	*ns*	*ns*	*ns*	<0.001	<0.001	<0.001	*ns*	<0.001
	*Tlr4*	*ns*	*ns*	<0.01	<0.05	*ns*	*ns*	<0.001	<0.001	*ns*	<0.001
	*Tlr9*	*ns*	<0.05	*ns*	*ns*	*ns*	*ns*	*ns*	<0.001	*ns*	<0.001
	*Myd88*	*ns*	<0.001	*ns*	*ns*	*ns*	<0.001	<0.001	ns	*ns*	*ns*
	*Irf3*	<0.001	<0.001	*ns*	<0.001	*ns*	*ns*	*ns*	*ns*	*ns*	*ns*
	*Irf7*	<0.001	<0.001	*ns*	*ns*	*ns*	<0.001	<0.001	ns	*ns*	*ns*
	*Tnfα*	*ns*	*ns*	*ns*	*ns*	<0.001	*ns*	*ns*	<0.001	*ns*	<0.001
	*Il6*	<0.001	<0.05	*ns*	*ns*	*ns*	<0.001	<0.01	*ns*	*ns*	*ns*
	*Ifnβ*	<0.001	<0.001	*ns*	*ns*	*ns*	<0.05	<0.05	*ns*	*ns*	*ns*
**Trigeminal ganglia**	*Tlr2*	*ns*	*ns*	<0.001	*ns*	<0.001	*ns*	*ns*	*ns*	*ns*	*ns*
	*Tlr3*	*ns*	*ns*	<0.001	*ns*	<0.001	*ns*	*ns*	*ns*	*ns*	*ns*
	*Tlr4*	*ns*	*ns*	<0.001	*ns*	<0.01	<0.01	<0.01	*ns*	*ns*	*ns*
	*Tlr9*	*ns*	*ns*	*ns*	<0.001	*ns*	*ns*	*ns*	*ns*	*ns*	*ns*
	*Myd88*	<0.001	<0.001	*ns*	*ns*	*ns*	<0.001	<0.001	*ns*	*ns*	*ns*
	*Irf3*	*ns*	*ns*	*ns*	*ns*	ns	*ns*	*ns*	<0.05	*ns*	*ns*
	*Irf7*	<0.001	<0.001	*ns*	*ns*	*ns*	<0.01	<0.001	*ns*	*ns*	*ns*
	*Tnfα*	*ns*	*ns*	*ns*	*ns*	*ns*	*ns*	*ns*	*ns*	*ns*	*ns*
	*Il6*	*ns*	*ns*	<0.001	*ns*	*ns*	*ns*	*ns*	*ns*	*ns*	*ns*
	*Ifnβ*	*ns*	*ns*	*ns*	*ns*	*ns*	*ns*	*ns*	*ns*	*ns*	*ns*
**Brain stem**	*Tlr2*	*ns*	*ns*	*ns*	*ns*	<0.01	*ns*	*ns*	*ns*	*ns*	*ns*
	*Tlr3*	*ns*	*ns*	*ns*	*ns*	*ns*	*ns*	*ns*	*ns*	*ns*	*ns*
	*Tlr4*	*ns*	*ns*	*ns*	<0.001	*ns*	<0.01	<0.01	*ns*	*ns*	*ns*
	*Tlr9*	*ns*	*ns*	*ns*	*ns*	<0.001	*ns*	*ns*	*ns*	*ns*	*ns*
	*Myd88*	<0.001	<0.01	*ns*	*ns*	*ns*	<0.001	<0.001	*ns*	*ns*	*ns*
	*Irf3*	*ns*	*ns*	*ns*	<0.001	*ns*	*ns*	*ns*	<0.05	*ns*	*ns*
	*Irf7*	<0.001	<0.001	*ns*	*ns*	*ns*	<0.001	<0.001	*ns*	*ns*	*ns*
	*Tnfα*	*ns*	*ns*	*ns*	*ns*	<0.001	*ns*	*ns*	*ns*	*ns*	*ns*
	*Il6*	*ns*	*ns*	*ns*	*ns*	<0.001	*ns*	*ns*	*ns*	*ns*	*ns*
	*Ifnβ*	*ns*	*ns*	*ns*	*ns*	<0.01	*ns*	*ns*	*ns*	*ns*	*ns*

P value: *** ≤0.001, ** ≤0.01, * ≤0.05, *ns*  =  not significant.

In the whiskers area of DA rats the increase of cell surface *Tlr2* expression was delayed starting from 2 dpi, while in PVG rats an increase was evident from 1 dpi. In DA rats increased expression of *Tlr2* was observed in the trigeminal ganglia at 2 dpi and in the brain stem at 4 dpi, while the expression in PVG rats was unchanged.

*Tlr3* expressed in cell endosomes recognizes double-stranded RNA. The levels of *Tlr3* were increased at 12 hpi and 1 dpi in the whiskers area of the DA rats, while differences were significant also at later time points in PVG rats. Baseline expression of *Tlr3* in naïve animals was higher in DA, than in PVG rats. Changes were significant in the trigeminal ganglia of the DA rats at 2 dpi and 4 dpi, whereas in the brain stem differences were not significant in any of the two strains.

*Tlr4* found on cell surfaces recognizes envelope proteins and lipopolysaccharides. Differences in the expression levels of *Tlr4* in the whiskers area of the DA rats were significant at 2 and 3 dpi and in PVG at 1, 2 and 4 dpi. In the trigeminal ganglia of DA rats changes were significant at 2 and 4 dpi and in the brain stem at 3 dpi.

Expression of endosomal *Tlr9* was elevated in the whiskers area of the DA rats at 1 dpi and in PVG from 2 dpi. *Tlr9* was up-regulated in trigeminal ganglia of DA rats from 4 dpi and from 3 dpi in the brain stem. No significant change was detected beyond the whiskers area in PVG rats.

The adaptor molecule *Myd88* is essential for the signaling cascade of most Toll-like receptors including *Tlr2*, *-4* and *-9*. *Myd88* expression was significantly increased in all compartments of both DA and PVG rats up to 1 dpi, but decreased to the control levels at later time points (Table 2).

Interferon regulatory factor 3 (*Irf3)*, which is a transcription factor involved in *Tlr* signaling and production of type I interferons, was significantly lower in the whiskers area of infected DA rats compared to naïve controls up to 3 dpi, while in the brain stem the expression was higher at 3 dpi.

In contrast, *Irf7* which is important for *Tlr9* signaling was increased significantly in all compartments of both DA and PVG rats up to 1 dpi.

The pro-inflammatory cytokines *Tnfα* and *Il6* are both induced by *Tlr* activation. *Tnfα* was increased in the whiskers area of DA rats at 4 dpi, while in PVG rats at both 2 dpi and 4 dpi. Changes were not significant over time in the trigeminal ganglia in any of the two strains, but DA rats had elevated expression at 4 dpi in the brain stem. *Il6* expression levels were significantly elevated in the whiskers area at 12 hpi and then declined in both strains. In the trigeminal ganglia of DA rats expression of *Il6* was increased at 2 dpi and in the brain stem at 4 dpi while expression did not change in the PVG rats in these two compartments.

The type I interferon *Ifnβ* expression was elevated in the whiskers area of both DA and PVG rats up to 1 dpi. In the brain stem of DA rats elevated levels were seen at 4 dpi.

In both strains the main changes detected in mRNA expression of the studied molecules after HSV-1 infection compared to naïve levels were found in the whiskers area. Notably, expression of *Tlr2, Tlr4, Myd88* and *Tnfα* were up-regulated at earlier time points in the resistant PVG rats, while in DA rats *Tlr9* was up-regulated earlier.

## Discussion

Innate immunity plays a critical role in the control of HSV-1 infection and early recognition of HSV-1 is crucial in the response of the host to prevent viral replication [6]. We have previously shown that subcutaneous injection of neurovirulent HSV-1 into the whiskers area caused lethal HSE in the susceptible DA strain, whereas in the resistant PVG strain no clinical manifestations were observed [5]. The underlying host determinants regulating HSE susceptibility are largely unknown and we wanted here to investigate the impact of the host innate immune reaction in a susceptible *vs.* a resistant rat strain to identify differences in the innate immunity regulating HSE.

The independent and/or parallel roles of *Tlr2* and *-9* in recognition of HSV-1 have been demonstrated *in vitro* [4]. Also, the synergistic role of *Tlr2* and -*9* was demonstrated in a HSV-2 encephalitis model in knock-out and double-knock-out mice [7]. TLR2 in patients has been identified as a host factor mediating cell entry of Cytomegalovirus by recognizing envelope glycoprotein gp B (gB) and gp H (gH) and subsequently initiate an inflammatory cytokine secretion that is independent of viral replication [8], [9]. *Tlr2* is associated with pro-inflammatory cytokine secretion, whereas *Tlr9* elicits also the secretion of type I *Ifn* (Figure 5K). Our results determine a host dependent difference of *Tlr2* and *-9* expression with a subsequent difference in *Tnfα,* but not in *Ifnβ* production suggesting that *Tlr2* is the receptor mediating the difference in virus spread seen at early time points.

At 12 hpi HSV-1 staining was similar in both strains surrounding the smaller nerve fascicles, was reduced at 1 dpi and only increased in DA rats with time. Thus, it is possible that the cell type infected and the different combinations of *Tlrs* activated may have a profound influence on the outcome of the infection.

In the resistant PVG rat expression of *Tlr2* and -*9* was significantly higher in the whiskers area at 1 and 2 dpi, the same time point at which the highest number of phagocytic cells was detected. Since *Tlr2* is found on the surface of macrophages and dendritic cells, it is likely that the increase in expression reflects a more rapid and vigorous recruitment of phagocyting cells in the resistant PVG strain. In peripheral nerves Schwann cells express high levels of TLRs, with TLR3 and -4 being the most prominent, whereas in sensory and motor neurons the levels are minimal [10]. In the central nervous system microglia, astrocytes, oligodendrocytes and neurons express TLR2, -3, -4 and -9 [11], [12]. A *Tlr2* signaling defect in DA rats could lead to impaired immune activation contributing to the spread/replication of HSV-1. Interestingly, *Tlr9* expression has also been associated with phagocytic capacity [13] and the increase in mRNA seen in PVG correlates with the increased infiltration of phagocytic cells seen in this strain compared to DA. Both the quantity and quality of the phagocytic response are thus implicated in the host protective response of PVG rats against HSE. Conversely, the slow induction of *Tlr2* and *-9* in parallel with the delayed infiltration of Iba1^+^/ED1^+^ cells in the whiskers area of DA rats is associated with susceptibility to HSE. Further studies are needed to dissect the relevant mechanisms and role of different immune cell populations more in detail.

We showed by mRNA expression analysis that immune activation started at the site of virus entry in the whiskers area in both DA and PVG rats. We have previously shown that in PVG rats HSV-1 infection did not penetrate beyond the level of the whiskers area [5]. Consequently, most markers of early innate immune responses remained quiescent in the PVG rats at the level of the trigeminal ganglia and in the brain stem. On the contrary, the expression of most studied targets became up-regulated in DA rats and increased over time in all the studied compartments. Thus, DA rats are susceptible to HSE despite mounting a more intense inflammatory reaction than PVG rats.

UNC-93B is a protein resident in the endoplasmic reticulum which regulates responses of intracellular TLRs and it specifically interacts with TLR3, -7 and -9 [14], [15]. It is known that genetic variability in the gene encoding the intracellular protein UNC-93B, important for TLRs mediated type I interferon production, increases susceptibility to HSE in humans, as well as deficiency in TLR3 [16], [17]. However, in our HSE model an elevated expression of *Tlr3* in the whiskers area was seen in the susceptible rat and we hypothesized that the susceptibility was not caused by low expression of *Tlr3*. In addition, the adaptor protein *Myd88* plays a major role in the *Tlr* signaling cascade and it has been shown previously that *Myd88^−/−^* mice were highly susceptible to lethal HSV encephalitis [18], [19]. Our data suggest that *Myd88* expression was not influencing the difference in susceptibility seen in DA and PVG rats.

*Irf7* which is required for *Tlr3*, *-4* and *-9* signaling, has been demonstrated to have a decisive role in HSV-1 infection in a mouse model [20]. We detected very high expression levels of *Irf7* in both DA and PVG rats within the first 24 hours after infection. However, in the susceptible DA rats the higher expression levels of *Irf7* compared to the PVG rats were not protective, indicating that it does not play a decisive in our rat model. *Myd88* and *Irf7* were the only markers that had early increased expression in PVG rats in the trigeminal ganglia and the brain stem, suggesting a retrograde or systemic immune reaction. In addition, DA rats expressed significantly higher levels of the pro-inflammatory cytokine *Tnfα* compared to PVG in all the anatomical compartments studied, however this was also found not to be protective against HSE. *Tnfα* is known to penetrate the perineurium [21] and the barrier role of the perineurial cells in neuroinflammation has been recently shown in a rat model [22], however the mechanism is not yet fully understood.

In our model we saw intense HSV-1 spread and replication in the perineurium of susceptible DA rats, while in the resistant PVG rats the viral levels in the whiskers area decreased over time and remained in the outer part of the epineurium. We identified an important temporal and a spatial difference in the spread of HSV-1 in DA compared to PVG rats. The strain differences in the distribution of HSV-1 in the whiskers area were associated with early recruitment of NK and CD8^+^ cells in the susceptible DA strain and more rapid recruitment of phagocyting cells in the resistant PVG strain. This indicates that the early recruitment of NK and CD8^+^ cells in DA rats was not sufficient to control the infection and could contribute to the subsequent progression to HSE. In a recent study it was shown that NK^+^, NK-T^+^, CD4^+^, CD8^+^ and γδT-cells individually did not restrict HSV-1 spread, but suggested a combinatory role of NK and CD8^+^ cells in regulating virus spread in HSE resistant BL/6 mice [23]. However, this was not corroborated in the rat strains examined here, since high NK and CD8^+^ cell activity and low phagocytic activity were correlated to HSE susceptibility.

In the susceptible DA rats less immune cells were recruited to the inner part of the epineurium which most likely facilitated the viral spread to the perineurial cell layer. Since the high density of phagocytic macrophages co-localised with HSV-1 in the perineurium did not prevent viral replication, it cannot be excluded that these cells possibly contributed to viral spread to the CNS. Collectively, these data indicate the importance of early recruitment of phagocyting cells and their ability to control the infection by preventing viral spread to perineurial cells and thereby restricting propagation of the virus along and in the nerves.

In conclusion, data provided herein demonstrated that an impaired host defense against HSV-1 infection in the susceptible DA strain with delayed infiltration of macrophages as well as early recruitment of NK and T cells to the infection site and reduced activation of *Tlr2* and *-9* influenced HSV-1 entry, replication and spread to the CNS. The distinct differences in host strain-dependent spread and replication of HSV-1 in the perineurial cell layer might support the hypothesis that the genetic properties of the perineurial cells could possibly have a key role in viral entry to the CNS and progression to HSE. Further investigations are needed to clarify the exact mechanisms of how HSV-1 enters the nerves and later the CNS after peripheral infection.

## Materials and Methods

### Animals

All experiments in this study were performed in accordance with the guidelines from the Swedish National Board for Laboratory Animals and the European Community Council Directive (86/609/EEC) and approved by the Swedish ethical committee (Stockholm's North Ethical Committee - Stockholms Norra Djurförsöksetiska Nämd) (ethical permit N340/08).

The experiments were carried out on inbred male Dark Agouti DA (RT1^av1^) originally obtained from the Zentralinstitut für Versuchstierzucht (Hannover, Germany) and MHC-identical Piebald Virol Glaxo PVG.1AV1 (RT1^av1^) (shortly referred to as PVG in this article) originally obtained from Harlan UK Limited (Blackthorn, UK). Rats were bred at the animal facility at Centre for Molecular Medicine, Karolinska University Hospital (Stockholm, Sweden).

All rats were 45 days old when taken into the experiment and virus-infected DA and PVG rats were analyzed at 5 dpi using immunohistochemistry and qRT-PCR. As controls, naïve animals at the age of 49 days, corresponding to 5 dpi in infected animals and Hank's solution-injected DA and PVG rats taken at 5 dpi were used (Table S1).

For qRT-PCR, 5 individuals of each strain DA and PVG were analyzed at each time point (12 hpi, 1, 2, 3 and 4 dpi) together with controls including 3 naïve and 3 Hank's solution injected rats of each strain (Table S1).

During the experiment animals were kept in a full-barrier animal facility, at the Astrid Fagræus laboratory, within the Swedish Institute for Infectious Disease Control (SMI), in groups of 3 to 5 animals per cage under specific pathogen-free and climate-controlled conditions, with 12 h light/dark cycles and ambient temperature of 21°C. The rats were housed in Eurotype IV Polystyrene cages, in enriched individually ventilated cage (IVC) system (Tecniplast, Italy) containing tin nests, aspen wood chips, aspen wood shavings and aspen chew blocks (Tapvei, Finland) and fed standard rodent chow (SDS, England) and water *ad libitum*.

Rats were anesthetized with 2% Isoflurane (Isoba® vet. Intervet AB, Sweden) before subcutaneous (*s.c.*) injection of HSV-1 or Hank's solution and before sacrifice by intraperitoneal (*i.p.*) injection of lethal dose of Pentobarbital vet. (Apoteket Produktion & Laboratorier AB, Sweden, 500 mg/kg).

### Virus

HSV-1 virus strain I-2762 was isolated from a diagnostic brain biopsy taken from a male patient on day 2 after onset of the first clinical symptoms of HSE. The patient died 2 days later, as a consequence of the infection. The virus isolation from the brain biopsy was approved by the Ethics committees at Karolinska Institutet, Göteborg, Linköping, Lund, Umeå and Uppsala Universities in 1981, as a part of the Swedish Multicentre Study on Acyclovir versus Vidarabine in Herpes simplex encephalitis [24] and has been conducted according to the principles expressed in the Declaration of Helsinki. The virus was propagated in green monkey kidney cells (GMK-AH1) for maximum two passages, suspended in Hank's Balanced Salt solution and was aliquoted and stored at −80°C. The isolate was typed as HSV-1 by enzyme-linked immunosorbent assay (ELISA) using type-specific monoclonal antibodies (mAbs) and infectivity titers were expressed in plaque forming units per milliliter (PFU/ml). In previous experimental studies this strain showed a high degree of neurovirulence and neuroinvasiveness both *in vivo* and *in vitro* [25], [5]. After being thawed to room temperature, 100 µl virus suspension, containing 2×10^6^ PFU HSV-1 was injected instantaneously subcutaneously (*s.c.*) into the area of the whiskers' base unilaterally, on the right side, under 2% Isoflurane anesthesia.

### Immunohistochemistry

Rats were perfused transcardiacally with 50 ml warm (+37°C) heparinized (10 I.U./ml Heparin LEO 5000 IE/KY/ml - LEO Pharma AB, Sweden) 0.9% saline solution, followed by 50 ml warm and then 200 ml cold (+4°C) fixative containing 4% paraformaldehyde and 0.4% picric acid in 0.16 M phosphate buffer (pH 6.9) [26], [27]. The whiskers' pad, the trigeminal ganglion and the brain stem were dissected from the ipsilateral side, immersed in the same fixative for 90 minutes and then cryoprotected in 0.1 M phosphate buffer containing sucrose, 0.02% sodiumazide (Sigma-Aldrich, Sweden) and 0.01% bacitracin (Sigma, Sweden). This procedure was carried out at SMI and the tissue samples were further processed at the Department of Clinical Neuroscience at Karolinska Institutet.

Fourteen micrometer (µm) thick sections were cut from the whiskers pad (coronal/transversal sections), the trigeminal ganglia (sagittal/longitudinal sections) and from the brain stem (coronal sections) at the level of the trigeminal nerve entry (Bregma -10.04) [28] in a cryostat (Microm, Heidelberg, Germany) and thawed onto Superfrost Plus® (Menzel, Germany) microscope glass slides. These were kept in −20°C freezer until processing. The sections were subjected to the indirect immunofluorescence staining [29] and incubated overnight in humidifying chambers at +4°C with following antibodies, diluted in phosphate-buffered saline (PBS) containing 0.3% Triton X-100, 0.5% BSA and 0.01% sodium azide (Sigma), covered with Parafilm M (Alcan Packaging, USA): rabbit anti-HSV-1 (1∶100) binding to HSV-1 type major glycoproteins in the HSV envelope (Dako Cytomation, Denmark), was used for visualizing virus-spread; rabbit anti-glial fibrillary acidic protein (GFAP) (1∶100) (Dako, Denmark) to stain astrocytes and Schwann cells [30], [31], [32]; mouse monoclonal anti-neuronal class III β-tubulin (Tuj1) (1∶500) (Covance, Germany) to visualise neurons/nerves; mouse monoclonal anti-O4 (1∶150) (Millipore, USA) recognizing oligodendrocyte marker O4 on cell bodies and processes of oligodendrocytes surrounding the axons in the peripheral tissue to visualize oligodendrocytes; rabbit anti-allograft Inflammatory Factor 1 (Iba1) (1∶200) (Wako Chemicals, USA) to visualize macrophages and microglia [33]; mouse monoclonal anti-complement receptor 3 (CD11b/c) (1∶200) (BD Pharmingen, Sweden) to visualize macrophages and granulocytes [34]; mouse monoclonal anti-ED1 (1∶200) (Serotec, Germany) to visualize lysosomal membrane of phagocyting cells; mouse monoclonal anti-MHC I, clone OX18 (1∶200) (Novus Biologicals, USA) to visualize expression of major histocompatibility class I molecules; mouse monoclonal anti-MHC II, clone OX6 (1∶200) (Serotec) to visualise major histocompatibility class II molecules; mouse monoclonal anti-CD8 (1∶200) (Serotec) to visualize cytotoxic T-cells and NK-cells and mouse monoclonal anti-NKRp1 (1∶200) (Harlan SeraLab, UK) to detect natural killer cells (NK).

After incubation with primary antibodies, sections were rinsed and incubated with Alexa Fluor™® 488 goat anti-rabbit (Molecular Probes, USA) or Alexa Fluor™® 594 goat anti-mouse (Molecular Probes) secondary antibodies. Primary and secondary antibodies used in this study are presented in Table S2.

A semi-quantitative assessment of Iba1^+^, MHC II^+^ and ED1^+^ cells was done by counting positive cells visually in the area surrounding the nerve fascicles (inner part of the epineurium) in the whiskers area of DA and PVG rats. Six different sections were analyzed for each animal and positive cells were counted in the area surrounding 5 nerve fascicles in the HSV-1 infected area per section. Micrographs were taken on a Zeiss Axioskop microscope system and processed in Adobe Photoshop CS3.

### Hematoxylin-eosin staining

Histological staining with Mayer's hematoxylin (Histolab) and eosin (Sigma-Aldrich) (H&E) was performed to visualize inflammatory infiltrations in the tissues and pathology caused by HSV-1 infection.

### Quantitative real-time PCR

All rats used for qRT-PCR (Table S1) were perfused transcardially with 50 ml warm (+37°C) heparinized (10 I.U./ml) 0.9% saline solution to flush the blood from the tissues. From each individual used for qRT-PCR, the whiskers pad, the trigeminal ganglion and the brain stem were dissected from the ipsilateral side, instantaneously frozen on dry ice and kept at −80°C until further processing.

Tissues were disrupted using Lysing Matrix D tubes (MP Biomedicals, Irvine, CA, USA) on a FastPrep homogenizer (MP Biomedicals) and mRNA was extracted using RNeasy mini-columns (Qiagen, West Sussex, UK) including 30 minutes on-column DNase digestion (Qiagen, 27 Kunitz units). cDNA was prepared by reverse transcription of 10 µl total mRNA using random hexamer primers (0.1 µg/ml; Gibco BRL, Invitrogen, Stockholm, Sweden) and Superscript Reverse Transcriptase (200 U; Gibco, BRL). Real-time PCR was performed using a BioRad iQ5 iCycler Detection System (BioRad Laboratories, Hercules, CA, USA) with a three-step PCR protocol (95°C for 3 minutes, followed by 40 cycles of 95°C for 10 seconds, 60°C for 30 seconds and 72°C for 30 seconds and 36 cycles of 55°C for 30 seconds), using SYBR green as fluorophore (BioRad Laboratories, Sweden). Primers were designed using the Beacon designer 5.0 (PREMIER Biosoft, USA) and the Primer Express (Perkin-Elmer) of ABI (Applied Biosystems, Sweden) software. Primer specificity was assessed by product evaluation on silver gel and by running a dissociation curve for each sample. Relative quantification of the mRNA levels was performed using the standard curve method using serial 5-fold dilutions from a pool of undiluted samples as standard. Relative amounts of mRNA were calculated as the ratio between the expression of the specific target and the expression of the housekeeping genes *Gapdh* and *Hprt* using the Bio-Rad iQ5 program v2. All tissue samples from each individual were analyzed in triplicates.

The following targets were analyzed: *Tlr2*, *Tlr3*, *Tlr4, Tlr9*, *Myd88*, *Irf3, Irf7*, *Tnfα*, *Il6* and *Infβ*. Primer sequences for target genes are shown in Table S3.

### Statistical analysis

Statistical analysis of qRT-PCR data and cell counts were performed using the GraphPad Prism 5.05 program (San Diego, CA, USA). The significance level of the differences between the DA and PVG strains over time were obtained by using two-way ANOVA with Bonferroni post-hoc test. The difference was calculated separately within each strain over time using one-way ANOVA with Bonferroni post-hoc test to show the significant changes among all observation groups. In Figure 5 we presented differences in expression between DA and PVG, while the differences in expression over time for each strain separately compared to the naïve tissue were described in the results and Table 2. A value of P≤0.05 was considered statistically significant.

## Supporting Information

Table S1 Number of rats used for the study (source: in-house breeding).
(0.03 MB DOC)

Table S2 Antibodies used for immunohistochemistry.
(0.04 MB DOC)

Table S3 Primers used for quantitative real-time PCR.
(0.04 MB DOC)
