# Supplementary material for: Influence of Perineurial Cells and Toll-Like Receptors 2 and 9 on Herpes simplex Type 1 Entry to the Central Nervous System in Rat Encephalitis
Source: PLoS One. 2010 Aug 27;5(8):e12350. doi: 10.1371/journal.pone.0012350 (PMC2929186; doi:10.1371/journal.pone.0012350)
Supplement: Table S1 — Number of rats used for the study (source: in-house breeding). (0.03 MB DOC) [file pone.0012350.s001.doc]

Table S1. **Nr. of rats used for the study (source: in-house breeding)**

|  | Time post-infection | | | | | | | | | | | | | | | |
| --- | --- | --- | --- | --- | --- | --- | --- | --- | --- | --- | --- | --- | --- | --- | --- | --- |
|  | **12 h** | | **1 dpi** | | **2 dpi** | | **3 dpi** | | **4 dpi** | | **Hank’s** | | **Naïve** | | **Total** |  |
| **Rat strain** | DA | PVG | DA | PVG | DA | PVG | DA | PVG | DA | PVG | DA | PVG | DA | PVG | DA + PVG |  |
| **qRT-PCR** | 5 | 5 | 5 | 5 | 5 | 5 | 5 | 5 | 5 | 5 | 5 | 5 | 3 | 3 | 66 |  |
| **Immunohistochemistry** | 3 | 3 | 3 | 3 | 3 | 3 | 3 | 3 | 3 | 3 | 3 | 3 | 3 | 3 | 42 |  |
