# Supplementary material for: Influence of Perineurial Cells and Toll-Like Receptors 2 and 9 on Herpes simplex Type 1 Entry to the Central Nervous System in Rat Encephalitis
Source: PLoS One. 2010 Aug 27;5(8):e12350. doi: 10.1371/journal.pone.0012350 (PMC2929186; doi:10.1371/journal.pone.0012350)
Supplement: Table S2 — Antibodies used for immunohistochemistry. (0.04 MB DOC) [file pone.0012350.s002.doc]

Table S2 **Antibodies used for immunohistochemistry**

| **Primary antibody** | | | | **Secondary antibody** | | | |
| --- | --- | --- | --- | --- | --- | --- | --- |
| **Ab** | **Conc.** | **Source** | **Cat. Nr.** | **Ab** | **Conc.** | **Source** | **Cat. Nr.** |
| Rb anti-HSV-1  (polyclonal) | 1:100 | DakoCytomation | B0114 | Alexa  Fluor^TM^® 488  go & rb | 1:200 | InVitrogen/  Molecular  Probes | A-11008 |
| Rb anti-GFAP  (polyclonal) | 1:100 | Dako | Z0334 | „ | „ | „ | „ |
| Rb anti-Iba1  (polyclonal) | 1:200 | WakoChemicals | 019-19741 | „ | „ | „ | „ |
| Mo anti-ED1  (CD68) (monoclonal) | 1:200 | Serotec | MCA341R | Alexa  Fluor^TM^® 594  go & mo | 1:200 | InVitrogen/  Molecular Probes | A-11005 |
| Mo anti-CD11b/c  (clone OX42) (monoclonal | 1:200 | BDPharmingen | 550299 | „ | „ | „ | „ |
| Mo anti-Tuj1  (monoclonal) | 1:500 | Covance | MMS-435P | „ | „ | „ | „ |
| Mo anti-O4  (monocl.) | 1:150 | Millipore | MAB345 | „ | „ | „ | „ |
| Mo anti-NKRp1  (monoclonal) | 1:200 | Harlan  Sera-Lab | MAB020 | „ | „ | „ | „ |
| Mo anti-MHC class I  (clone OX18)  (monoclonal) | 1:200 | NovusBiologicals | NB 120-  6405 | „ | „ | „ | „ |
| Mo anti-CD8  (monoclonal) | 1:200 | Serotec | MCA48G | „ | „ | „ | „ |
| Mo anti-MHC classII  (clone OX6) (monoclonal) | 1:200 | Serotec | MRCOX-6 | „ | „ | „ | „ |
