# Supplementary material for: Influence of Perineurial Cells and Toll-Like Receptors 2 and 9 on Herpes simplex Type 1 Entry to the Central Nervous System in Rat Encephalitis
Source: PLoS One. 2010 Aug 27;5(8):e12350. doi: 10.1371/journal.pone.0012350 (PMC2929186; doi:10.1371/journal.pone.0012350)
Supplement: Table S3 — Primers used for quantitative real-time PCR. (0.04 MB DOC) [file pone.0012350.s003.doc]

Table S3 **Primers used for quantitative real–time PCR**

| **Target** | **Forward** | **Reverse** | **Source** |
| --- | --- | --- | --- |
| *Tlr2* | CCAGAGGACTCAGGAGCAG | CACACACCAGCAGCATCAC | Eurofins MWG/  Operon |
| *Tlr3* | TTTCTCCCTAAAGGTCAGTTTATG | AATCAGACATTTCCTTCCATCC | “ |
| *Tlr4* | GCATCTGGCTGGGACTCTG | GATCTAGGTTCTTGGTTGAATAAGG | “ |
| *Tlr9* | ATGCCTTCGTGGTGTTCGAT | CCGCACTCGAAGCTCGTTAT | “ |
| *Myd88* | CAGAGTGGAGAGCAGTGTC | GGGCAGTAGCAGATGAAGG | “ |
| *Irf3* | TGGCTGACTTTGGCATCTTC | GCTAATCGCAACACTTCTTTCC | “ |
| *Irf7* | GCAAGTCATCAGAGTAGTAGCAC | AAGAGCCAGTCTCCGAACAG | “ |
| *Tnfα* | GACCCTCACACTCAGATCATC TTT | ACGCTGGCTCAGCCACTC | “ |
| *Il6* | GAAAAGAGTTGTGCAATGGCAA | ATT TCTGGAAGTTTCAGATTGTTTCT | Eurofins MWG/  Operon |
| *Ifnβ* | CTTTGCCATTCAAGTGATGCTC | ACAATAGTCTCATTCCACCAGTG | CyberGene AB |
| *Hprt* | CTCATGGACTGATTATGGACAGGAC | GCAGGTCAGCAAAGAACTTATAGCC | “ |
| *Gapdh* | TCAACTACATGGTCTACATGTTCCAG | TCC CAT TCT CAG CCT TGA CTG | “ |
